# Simultaneous Cross-Linking and Nanoparticle Anchoring by Dialdehyde Cellulose in Injectable Composite Chitosan/Polypyrrole Hydrogels

**DOI:** 10.1021/acsabm.5c02494

**Published:** 2026-02-19

**Authors:** Monika Muchová, Lukáš Münster, Roman Kolařík, Zdenka Víchová, Ondřej Vašíček, Petr Humpolíček, Jan Vícha

**Affiliations:** † Centre of Polymer Systems, 48362 Tomas Bata University in Zlín, tř. Tomáše Bati 5678, Zlín 760 01, Czech Republic; ‡ Institute of Biophysics of the Czech Academy of Sciences, Kralovopolská 135, Brno 612 00, Czech Republic; § Department of Fat, Surfactant and Cosmetics Technology, Faculty of Technology, Tomas Bata University in Zlín, nám. T. G. Masaryka 5555, Zlín 760 01, Czech Republic

**Keywords:** injectable, chitosan, dialdehyde cellulose, polypyrrole, composite hydrogels

## Abstract

The injectable composite hydrogel with covalently bound polypyrrole (PPy) has been prepared using dialdehyde cellulose (DAC) as a bifunctional cross-linker, forming dynamic imine bonds with water-soluble half acetylated chitosan (SCN) and simultaneously tethering the PPy nanoparticles by aldol condensation. The novelty lies in translating this dual chemistry into an injectable, self-healing hydrogel system, for the first time fully utilizing dynamic Schiff base cross-linking in combination with covalent PPy anchoring. PPy is also involved both in hydrogel cross-linking, altering its rheological behavior, but also providing antioxidative and anti-inflammatory effects. The resulting hydrogels exhibited shear-thinning behavior, rapid self-healing, and storage moduli ranging from 25 to 47 Pa, allowing for injection through 21 G needles. All formulations were noncytotoxic toward NIH/3T3 fibroblasts and RAW 264.7 macrophages. In scratch assays, SCN_DAC_20_PPy significantly accelerated wound closure, with the residual wound area to 39 ± 2% after 10 h versus 83 ± 7% for controls and 65 ± 3% for the corresponding PPy-free hydrogel. In LPS-stimulated macrophages, all hydrogels decreased nitric oxide production, and PPy-containing hydrogels additionally reduced IL-6 secretion. The SCN/DAC/PPy injectable hydrogels thus exhibit cytocompatibility, self-healing properties, and anti-inflammatory activity, representing a promising platform for the future development of advanced wound dressings.

## Graphical abstract

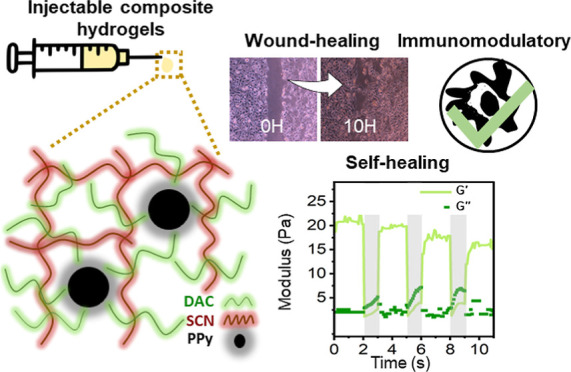

## 1 Introduction

Injectable hydrogels are widely used in the field of biomedicine, primarily for tissue regeneration,^1–3^ wound healing,^4^ drug delivery, or biosensors.^5,6^ Various polysaccharides are currently being investigated as matrices for injectable hydrogels, including hyaluronic acid, dextran, chondroitin sulfate, sodium alginate, Gellan gum, and chitosan.^7,8^

Chitosan, a partially deacetylated derivative of chitin containing β-(1 → 4)-linked d-glucosamine and *N*-acetyl-d-glucosamine, has wide applications in drug delivery, gene therapy, tissue engineering, and wound dressings due to its biocompatibility, biodegradability, antibacterial properties, and low toxicity.^4,8,9^ However, the insolubility of chitosan under physiological conditions limits its use in certain biomedical applications, such as injectable formulations, where the use of an acidic solvent is not feasible.^8^ One solution to this problem has been the development of various synthetic water-soluble derivatives of chitosan, such as trimethyl chitosan, carboxymethyl chitosan, and succinyl chitosan, among others.^8,10^ These derivatives are soluble at physiological pH, albeit at the cost of artificial modifications to chitosan. Such modifications may result in nonphysiological degradation products, whose fate in the body and long-term effects are largely unknown.

An alternative, yet rather underutilized, approach is to use water-soluble half acetylated chitosan (soluble chitosan, SCN). SCN is soluble at physiological pH without artificial functional groups.^11,12^ It is prepared by controlled deacetylation of chitin or partial reacetylation of chitosan with a high degree of deacetylation (DD) (e.g., 70–85%). The only modified parameter is thus the DD, a native property of chitosan. These modifications are thus completely natural as they involve no synthetic groups being grafted to chitosan, unlike trimethyl chitosan and other derivatives. The solubility is achieved simply due to a partial disruption of the ordered hydrogen bridge network^12^ by reducing the DD to approximately 50%. This is caused by the presence of randomly distributed amino and *N*-acetyl groups, which prevent the formation of larger solubility-limiting crystallites.

To obtain injectable chitosan-based hydrogels, chitosan has to be cross-linked through dynamic covalent bonding (a reversible Schiff base reaction), which allows the hydrogels to liquefy under shear stress during injection and then return to their original gel state after the stress is removed, enabling an effective self-healing process.^4^ This can be achieved using dialdehydes such as glutaraldehyde. However, glutaraldehyde is highly toxic and may be released from the hydrogel during shear-thinning/gelation stages, which poses a problem for clinical applications.^8^ Instead, dialdehyde cellulose (DAC), prepared by regioselective oxidation of cellulose, can be used.^13^ DAC has significantly lower toxicity than glutaraldehyde,^14^ is biodegradable in vivo,^15^ and has higher cross-linking efficiency at low concentrations due to its ability to react with many macromolecules simultaneously.^16^ The use of DAC as a cross-linker for classical DAC/chitosan hydrogels has been investigated by Kim et al.,^17^ while the synthesis of injectable hydrogels with chitosan derivatives, such as carboxymethyl chitosan^18^ or quaternized chitosan,^19^ or in acidic mixtures of highly deacetylated chitosan^20^ was discussed by others. The DAC as a cross-linker for chitosan is thus well-known.

There is, however, another, much less known aspect of DAC chemistry that was only recently discovered by us.^21^ It is the ability to spontaneously bind pyrrole and polypyrrole (PPy) through aldol condensation reaction.^21–23^ PPy is an intrinsically conductive polymer that has attracted considerable attention as a bioactive component of wound dressings and tissue-engineering scaffolds. Beyond its electronic conductivity under physiological conditions, PPy can scavenge reactive oxygen species, attenuate pro-inflammatory signaling, and modulate cell adhesion and migration, which collectively support tissue repair.^24–26^ PPy-based materials have been reported to accelerate re-epithelialization, enhance angiogenesis, and reduce bacterial burden in cutaneous wounds even without external electrical stimulation.^12,27,28^ Colloidal PPy^26^ may thus act as a multifunctional additive, providing anti-inflammatory functions and modulating the behavior of fibroblasts and macrophages in favor of efficient wound closure, even without external electrical stimulation. Adding well-defined, prefabricated PPy nanoparticles also enables precise control over PPy loading and dispersion in hydrogel, eliminating uncontrolled formation of PPy deposits during in situ pyrrole oxidation and contamination by residual oxidants.

A single biobased cross-linker, the DAC, can thus serve a dual function, simultaneously binding water-soluble half acetylated chitosan and incorporating PPy nanoparticles into the hydrogel network (see Scheme 1)^21^ without the need for further complex synthetic modifications, which are normally required for covalent PPy binding and would preclude the preparation of shear-thinning hydrogels.^29^

**Scheme 1:**
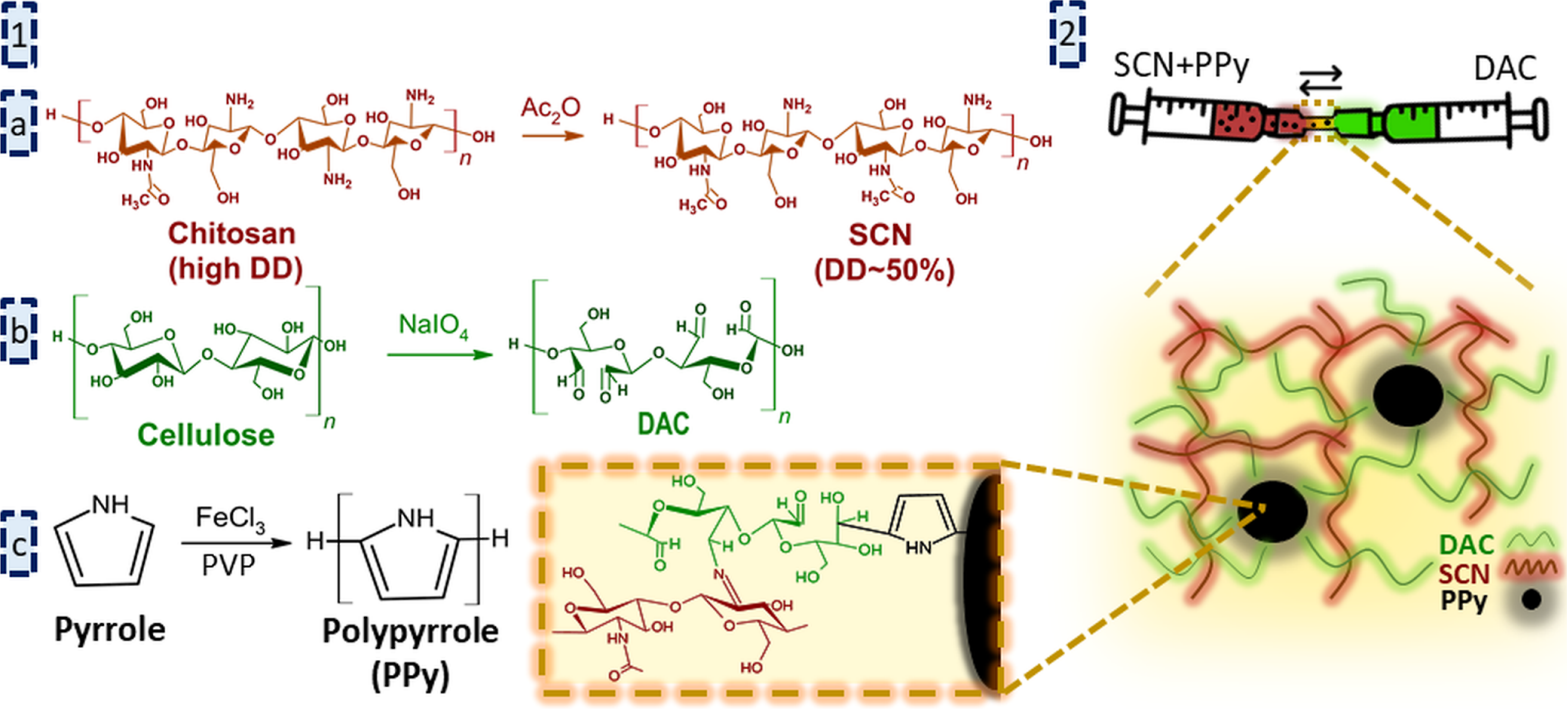
(1) Synthesis of (a) Soluble Half Acetylated Chitosan (SCN), (b) Dialdehyde Cellulose (DAC), and (c) Polypyrrole (PPy); (2) Schematic Illustration of Mixing SCN, PPy, and DAC Solutions Using Syringes with a Schematic Representation of the SCN_DAC_PPy Hydrogel Network and Binding Combining Schiff Bases (between DAC and SCN) and Aldol Condensation between DAC and PPy

While earlier studies on PPy composites focused on rigid nanofibrous mats, films, or the investigation of reaction mechanisms of DAC–PPy coupling,^12,21–23^ current work is the first to fully utilize dynamic Schiff base cross-linking in combination with covalent PPy anchoring to achieve shear-thinning, injectability, and rapid self-healing properties in a composite hydrogel. The novelty of this study thus lies in translating the dual chemistry of DAC into an injectable, self-healing hydrogel system, which would not be possible with classical methods of PPy anchoring.^29^ Such material allows addressing irregular wounds not only on the skin surface but possibly also within the body, greatly expanding the utility of this approach compared to earlier works. To the best of our knowledge, this is the first report of injectable self-healing composite hydrogels with covalently bound PPy. This article aims to explore the synthesis, material characterization, and basic biological tests to demonstrate the potential of this new approach.

## 2 Methods

### 2.1 Materials

Pyrrole (Sigma-Aldrich Co.), iron­(III) chloride (Sigma-Aldrich), poly­(vinylpyrrolidone) (PVP; Fluka, K 90, molecular weight *M*_w_ = 360 kDa), SigmaCell type 20 cellulose (*M*_w_ = 76 kDa, degree of polymerization DP = 468, *D̵* = 4.7; S3504, Sigma-Aldrich Co.), sodium periodate (NaIO_4_; Penta, Czech Republic), ethylene glycol (Penta, Czech Republic), chitosan low molecular weight (*M*_w_ = 50–190 kDa, according to the supplier; Sigma-Aldrich Co.), glacial acetic acid (CH_3_COOH; Sigma-Aldrich Co.), absolute ethanol (Et–OH; VWR, Czech Republic), acetic anhydride (Ac_2_O), and sodium hydroxide (NaOH; Sigma-Aldrich Co. and Penta, Czech Republic) were used. All reagents were of analytical grade and applied without further purification.

### 2.2 Preparation of SCN, DAC, and PPy

Water-soluble half acetylated chitosan was prepared according to the procedure published by Qin et al.^30^ Initially, 2.2 g of chitosan was dissolved in 70 mL of 10% acetic acid. Subsequently, a mixture of 50 mL of ethanol containing 0.519 mL of Ac_2_O was added dropwise to the solution, and the mixture was stirred for 15 h at 40 °C. After this period, the solution was diluted by adding 50 mL of ultrapure water (UPW), and the pH was adjusted to 8.5 using a 5 M NaOH solution, resulting in the formation of gel particles. The mixture was then dialyzed for 72 h. Following this, the pH was adjusted to 6.5, leading to the dissolution of the gel particles. The mixture was purified by dialysis against UPW (72 h) and 0.1 M NaCl solution (24 h), followed by short dialysis (2 h) against UPW to remove salts. The product was then centrifuged (10,000 rpm, 10 min), filtered, and lyophilized. The DD was determined by nuclear magnetic resonance (NMR) spectroscopy to be 46% based on intensities of H2 from the d-glucosamine unit and CH_3_ from the *N*-acetyl-d-glucosamine.^31^

To prepare DAC, cellulose was oxidized using a 1.2 M excess of NaIO_4_ at 30 °C without light for 72 h, as described in the literature.^14,16^ Subsequently, the reaction was stopped with ethylene glycol, and the resulting product was purified using several cycles of centrifugation and homogenization. The resulting DAC suspension was then solubilized at 80 °C under a reflux condenser for 2 h. This was followed by purification of the solution through centrifugation, filtration, and 48 h of dialysis, and the final product was lyophilized. The degree of oxidation of the resulting soluble DAC was found to be 94 ± 2% based on oxime reaction and alkalimetric titration.^16^ The *M*_w_ of DAC was determined by GPC with a RALS/LALS detector to be 7600 Da, *D̵* = 1.13. The relatively low *M*_w_ of DAC is caused by the solubilization step as described earlier.^32^

For the synthesis of polypyrrole colloids (PPy), the PVP solution was prepared by dissolving 2 g of PVP in 25 mL of UPW and mixed with 0.335 g of pyrrole (5 mmol) dissolved in 25 mL of UPW, following earlier work.^12,26^ The mixture was sonicated for 30 min to obtain a homogeneous solution, which was then mixed with 50 mL of iron chloride solution (1.352 g; 5 mmol) in UPW. The mixture was left undisturbed at room temperature for 24 h. The obtained colloidal dispersions of polypyrrole (PPy) were transferred into a dialysis tubing (Spectra/Por 1, Spectrum Medical Instruments, USA; molecular weight cutoff of 7 kDa) and dialyzed against 0.2 M hydrochloric acid, which was later replaced by water. This process aimed to eliminate any residual monomer and oxidizing agents from the solution.^26^ The final concentration of the PPy colloidal particles was 21.9 mg/mL.

### 2.3 Fabrication of Injectable Hydrogels

To prepare injectable hydrogels, SCN and DAC were weighed in the ratios specified in Table 1. SCN was dissolved overnight at 45 °C. DAC was dissolved at 70 °C for 10–15 min in two different concentrations, corresponding to 10 and 20% of mol equivalent relative to SCN. After dissolution, a volume of 2.5 mL was withdrawn from each solution using a pipet designed for viscous solutions and transferred to a capped syringe. Syringes were subsequently connected by a silicon tube, and both solutions were thoroughly mixed by transferring the forming hydrogel from one syringe to the other. After mixing, the syringes with the prepared hydrogel were left to equilibrate overnight. For samples containing PPy, 86 μL of PPy colloid was subsequently added to the SCN (5 wt % of colloidal PPy particles relative to SCN) before mixing with DAC. The amount of PPy was selected based on an earlier study, which showed that higher fractions provided only limited benefits.^12^ The exact composition of the four prepared samples (two neat hydrogels and two containing 5 wt % of PPy) and their designation are given in Table 1.

**Table 1:** Preparation of SCN_DAC (10% or 20%) and Their Variants with PPy Colloidal Particles

sample	concentration of SCN (mg/mL)	volume (mL)	molar fraction of DAC (mol %)	SCN weight (mg)	DAC weight (mg)	PPy volume (μL)	total concentration of SCN (mg/mL)	total volume (mL)
SCN_DAC_10%	15	2.5	10%	37.5	3.7	-	7.5	5
SCN_DAC_20%	15	2.5	20%	37.5	8.2	-	7.5	5
SCN_DAC_10%_PPy	15	2.5	10%	37.5	3.7	86	7.5	5
SCN_DAC_20%_PPy	15	2.5	20%	37.5	8.2	86	7.5	5

### 2.4 Sample Characterization and Instrumentation

#### 2.4.1 Infrared Spectroscopy

An FT-IR spectrometer (Nicolet 6700, Thermo Fisher Scientific, USA) equipped with a diamond crystal and an ATR mode was used. The spectra were collected in the wavenumber range between 4000 and 400 cm^–1^, accumulating 64 scans per sample at a resolution of 4 cm^–1^.

#### 2.4.2 Gel Permeation Chromatography

A Waters HPLC Breeze chromatographic system (Waters, USA) equipped with a refractive index detector Waters 2414 (drift tube *T* = 60 °C) and a Tosoh TSK gel GMPWXL column (300 mm × 7.8 mm × 13 μm, column *T* = 30 °C) and a MalvernViscotek RALS/LALS detector were used. The mobile phase was 0.1 M NaNO_3_, *n* = 1.3340. The flow rate of 0.8 mL/min, injection volume 0.1 mL, d*n*/d*c* = 0.1350.

#### 2.4.3 Nuclear Magnetic Resonance

A JEOL 400 MHz NMR spectrometer (JEOL, Japan) has been used to determine the DD of the source chitosan and SCN sample. 10 mg of chitosan or SCN was dissolved in 0.5 mL of 0.1 M DCl. The DD was determined by comparing the intensity of the CH_3_ signal from the *N*-acetyl glucosamine unit (CH_3_ *N*-ac) and the signal of −C**H**(NH_2_) (H2) from the d-glucosamine unit. Spectrum assignment is based on Kassai.^31^

#### 2.4.4 X-ray Photoelectron Spectroscopy

An Axis Ultra DLD spectrometer, equipped with a monochromatic Al Kα source (*h*ν = 1486.7 eV), was operated at 75 W (5 mA, 15 kV) under a base pressure of ∼2 × 10^–8^ Pa, with a Kratos charge neutralizer. High-resolution spectra were acquired with a 0.1 eV step size and a pass energy of 20 eV, and an area of 300 × 700 μm was analyzed. Data were processed in CasaXPS (v2.3.15); all spectra were charge-referenced to the C 1s main component (C–C/C–H) at 285.0 eV, and a Shirley background was applied throughout. X-ray photoelectron spectroscopy (XPS) spectra were measured on dry-out thin films prepared by mixing and casting of main components (*n*_NH_2__/*n*_–CHO_ = 1:1) with and without addition of PPy (*m*_DAC_/*m*_PPy_ = 1:2).

#### 2.4.5 Dynamic Light Scattering Analysis

Zeta potential (ζ) and hydrodynamic radius of the PPy diluted solution were determined by dynamic light scattering (DLS) on a Zetasizer Nano ZS90 instrument (Malvern Instruments, UK) at 25 °C using the Smoluchowski model.^33^

#### 2.4.6 Rheological Analysis

Rheological analyses were conducted using a rotational rheometer Anton Paar MCR 502 (Anton Paar, Austria) with a parallel-plate measuring system consisting of an upper geometry with a roughened (sandblasted) surface and a diameter of 15 mm and a bottom plate equipped with sandpaper to prevent the hydrogel from slipping during measurement.

To study the viscoelastic properties of the hydrogel, rheological measurements were performed over an angular frequency range of 1 to 10 rad/s at a constant deformation of 1%. Time-sweep experiments with cyclic strain were conducted at an angular frequency of 1 Hz to examine the properties of the injectable and self-healing hydrogels. Thus, the magnitude of the amplitude of oscillatory deformation was gradually changed from low deformation (at 0.2% strain) for 2 min to high deformation (1000% strain) for 1 min for each interval. Additionally, rheology properties were measured at a deformation of 0.2% over a range of angular frequencies from 1 to 50 rad/s to characterize the material behavior under shear-thinning. Then, the strain-dependent rheological measurement of the prepared hydrogels was measured in the range from 0.1% to 1000% at a constant frequency (1 Hz).

#### 2.4.7 SEM Analysis

Images of dried hydrogel samples were obtained using a Nova NanoSEM 450 scanning electron microscope (FEI, Czech Republic) operated at a 5 kV accelerating voltage. All samples were sputtered by gold–palladium nanoparticles to prevent the charge accumulation effect. SEM micrographs are given in the Supporting Information.

#### 2.4.8 Conductivity Measurements

The resistivity of the prepared hydrogels was measured using the van der Pauw four-electrode method with a digital electrometer (Keithley 6517B), a voltage source (Keithley 2410), and a scanner (Keithley 7002). Measurements were performed by using a built-in Alternating Polarity Method. Briefly, a positive voltage is applied first; the current is measured after a specified delay (measure time); then, the polarity is reversed, and the current is measured again with the same delay. This process is repeated continuously, and the resistance is calculated based on a weighted average of the four most recent current measurements (three initial iterations are discarded to limit the effect of background currents). The conductivity of the samples was then calculated as the reciprocal of the resistivity, i.e., σ = 1/ρ, where ρ is the resistivity [S·cm^–1^]. Measurements were conducted in the swollen state at 25 °C. UPW with resistivity >18.2 MΩ·cm was used for hydrogel sample preparation.

#### 2.4.9 Evaluation of Cytocompatibility, In Vitro Wound Healing Promotion, and Immunomodulatory Activity

Detailed experimental protocols are provided in the Supporting Information (see Section S1). The cytocompatibility of hydrogel extracts was evaluated using NIH/3T3 fibroblasts according to ISO 10993-12. Wound healing capacity was investigated via scratch assay, and immunomodulatory effects were evaluated in RAW264.7 macrophages by analyzing cell viability, nitric oxide production, and IL-6 secretion following LPS stimulation.

#### 2.4.10 Statistical Analysis

Data are presented as means ± SEM. All assays were performed in quadruplicate, unless stated otherwise in the text. The data from the measurements were normalized to the reference in each experiment to account for the variability of the individual cell passages. Statistical analysis was performed using GraphPad Prism version 6.01 for Windows. Statistical differences were tested by one-way ANOVA, which was followed by Dunnett’s multiple comparison test.

## 3 Results and Discussion

### 3.1 Characterization of SCN, DAC, and PPy

The SCN prepared as described in Section 2.2 was characterized by FT-IR and NMR (Figure 1). Comparison of the FT-IR spectra of SCN with the source chitosan reveals an increased intensity of signals corresponding to amide groups, particularly the Amide I (1640 cm^–1^) and Amide II (1530 cm^–1^) bands in the SCN spectrum, confirming partial (re)­acetylation of the source chitosan. NMR analysis of the ^1^H spectrum revealed that the DD of SCN was 46%, which is a significant increase compared to the source chitosan (DD = 67% according to the same method, see Figure S1 for the ^1^H NMR spectrum). The solubility tests revealed that the prepared SCN was soluble up to 15 mg/mL in both water and PBS at pH 7.

**Figure 1:**
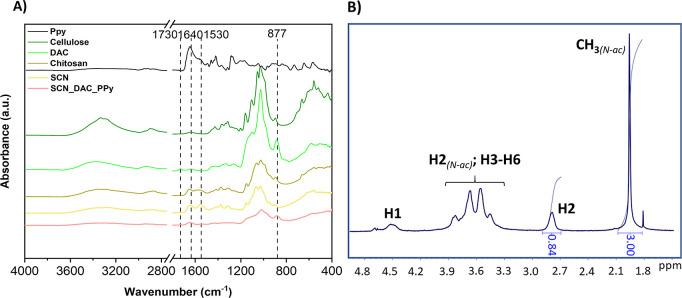
(A) FT-IR spectra of PPy, cellulose, DAC, chitosan, SCN, SCN_DAC_PPy, and (B) ^1^H NMR spectrum of SCN.

Cellulose was oxidized to DAC according to previous works.^14,16^ The FT-IR spectrum (Figure 1) confirmed the oxidation of cellulose to DAC, as evidenced by the formation of a weak band at around 1730 cm^–1^ (C=O group vibrations) and a band at 877 cm^–1^ (hemiacetal C–O–C vibrations), as shown in Figure 1.

PPy colloids prepared as described in Section 2.1 were characterized by FT-IR (Figure 1), showing characteristic vibrations of C=C bonds of conjugated pyrrole rings in the range of 1440 to 1560 cm^–1^ and bending vibrations of C–H bonds between 800 and 1000 cm^–1^.^12,26^ According to DLS, the hydrodynamic diameter of PPy colloids was 189 ± 3 nm, with a polydispersity index of 0.23 ± 0.01, and the zeta potential was 4.3 ± 0.5 mV.

Next, the ability of DAC to simultaneously form Schiff bases and react with PPy has been investigated by using XPS on dry-out thin samples. The XPS spectra of the dried mixture of SCN and DAC without PPy are shown in Figure 2A,C, and their PPy-containing equivalents are shown in Figure 2B,D.

**Figure 2:**
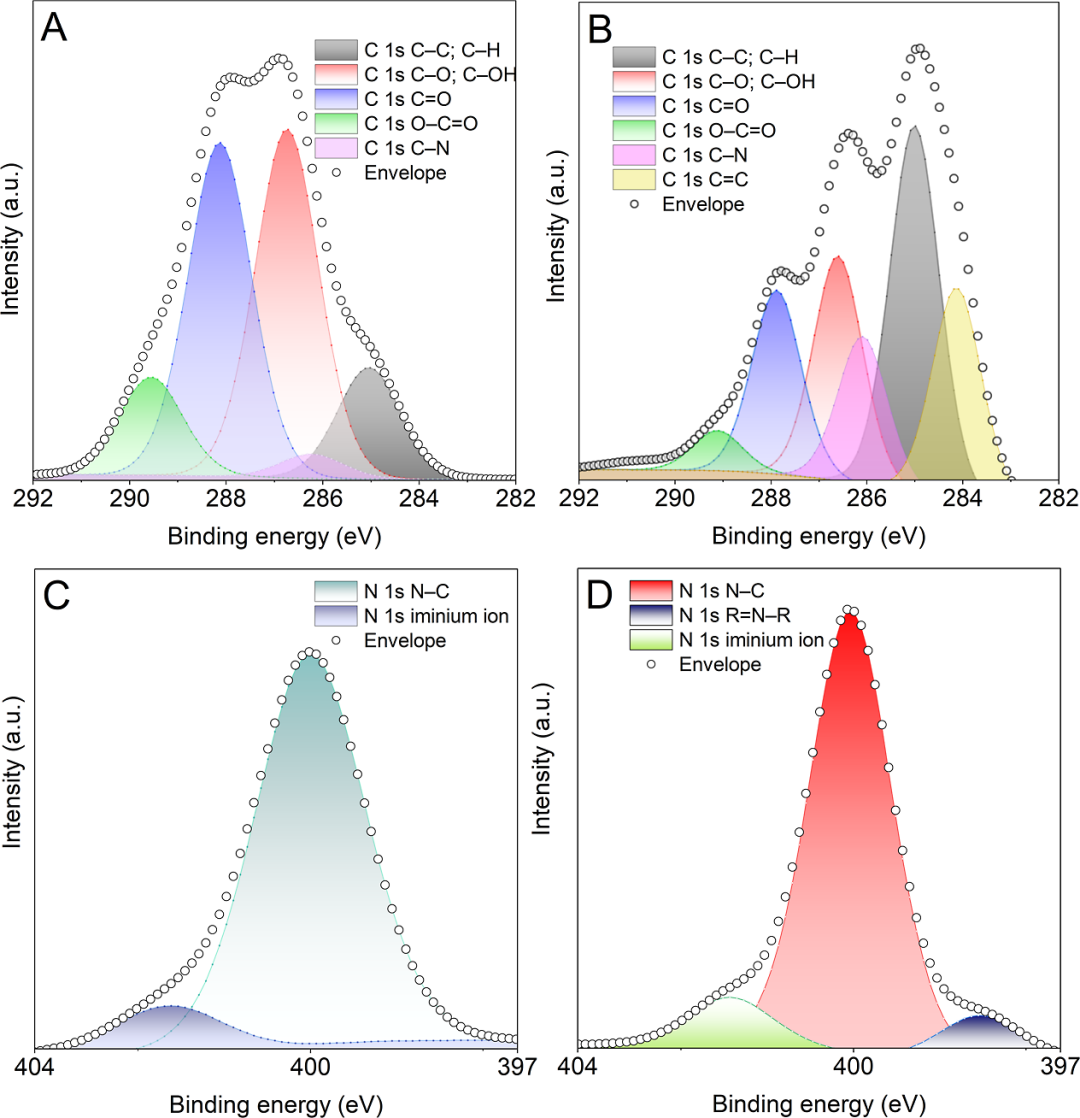
XPS spectra of (A) C 1s of sample without Ppy, (B) C 1s of sample with PPy, (C) N 1s of sample without PPy, and (D) N 1s of sample with PPy.

The C 1s region of the sample without PPy (Figure 2A) exhibited signals characteristic of the chitosan/DAC mixture, i.e., those belonging mainly to the C–C, C–OH, and C=O bonds. The N 1s region of the same sample in Figure 2C contains, besides the major signal of N–C bonds from chitosan, also a signal of the iminium ion (protonated imine bond) at 402.0 eV. This is the result of Schiff-base formation between the NH_2_ groups of chitosan and the aldehyde groups of DAC.

The presence of PPy in the second sample (right column in Figure 2) results in an increase in the intensity of C–C and C–N signals and the appearance of a new signal belonging to C=C at 284.1 eV in the C 1s region. The increase in the intensity of the C–OH signal relative to C=O (compared to Figure 2A) agrees with the expected aldol condensation reaction, where part of the C=O groups react with the end groups of PPy chains, forming PPy–CH­(OH)–DAC bonds, as shown in Figure 1. The presence of PPy also results in the appearance of the signal C=N–C from pyrrole cycles at 398.2 eV, along with an increased intensity of the C–N signal. The iminium ion signal in Figure 2D confirms the presence of Schiff bases also in the PPy-containing samples.

### 3.2 Characterization of Hydrogels

Four hydrogel samples were prepared as described in Section 2.3 (see Table 1 for more details). Besides neat hydrogel samples with 10 and 20 mol % of DAC relative to SCN (SCN_DAC_10 and SCN_DAC_20), two samples containing 5 wt % of PPy relative to SCN (SCN_DAC_10_PPy and SCN_DAC_20_PPy) were prepared.

#### 3.2.1 Rheological Measurements

The storage (*G*′) and loss (*G*″) moduli, complex modulus (G*), and damping factor (tan δ) were measured for prepared injectable hydrogel samples at different angular frequencies, as depicted in Figure 3.

**Figure 3:**
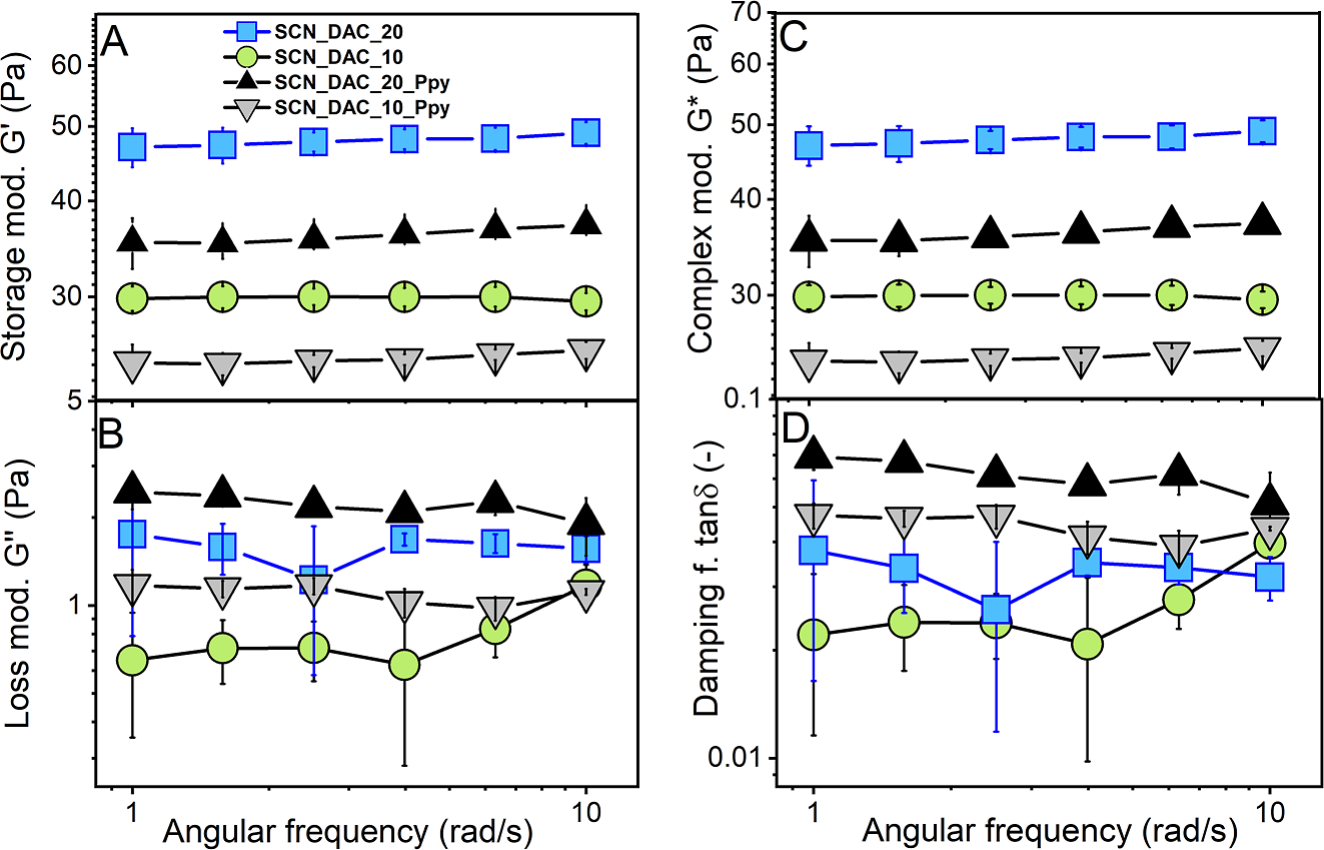
Storage (A), loss (B), and complex moduli (C), along with the damping factor (D) of SCN_DAC_10, SCN_DAC_20, SCN_DAC_10_Ppy, and SCN_DAC_20_PPy.

All samples exhibited a dominance of *G*′ over *G*″, indicating a permanent hydrogel-like structure. The values of storage modulus *G*′ (Figure 3A) ranged from approximately 25 Pa for the SCN_DAC_10_PPy sample to 47 Pa for SCN_DAC_20. The highest *G*′ value was observed for the sample labeled SCN_DAC_20, as expected due to its highest concentration of the DAC cross-linker. Upon addition of colloidal PPy, the *G*′ value decreased to approximately 35 Pa for the SCN_DAC_20_PPy sample. PPy colloidal particles likely bind part of the cross-linker to themselves, which limits its availability for cross-linking of the bulk hydrogel, resulting in a locally dense network topology, see below.^34^ A similar trend was observed for samples SCN_DAC_10 and SCN_DAC_10_PPy, where the *G*′ value decreased from ∼30 to around 25 Pa. The values of the loss modulus *G*″ (see Figure 3B) ranged from approximately 0.7 to 2.4 Pa. The complex modulus values (Figure 3C) correlated with the storage modulus values within experimental error. The damping factor tan θ of all samples was similar and is given in Figure 3D.

The storage moduli of 25–47 Pa observed for the investigated hydrogels fall within the range reported for soft, injectable wound-healing matrices designed to conform to irregular wound beds rather than to bear substantial mechanical loads. For example, chitosan and hyaluronic acid-based injectable hydrogel with comparable moduli have been shown to support re-epithelialization and regeneration of abdominal tissues in vivo.^35^ The relatively low modulus is therefore not expected to limit the applicability of these hydrogels as a topical wound filling material.

The shear-thinning and self-healing abilities of the prepared hydrogels were subsequently verified using various rheological measurements. As shown in Figure 4A, the viscosity of all prepared hydrogels decreased with increasing angular frequency, demonstrating characteristic shear-thinning behavior. This indicates excellent injectability properties of the prepared hydrogels.^36^

**Figure 4:**
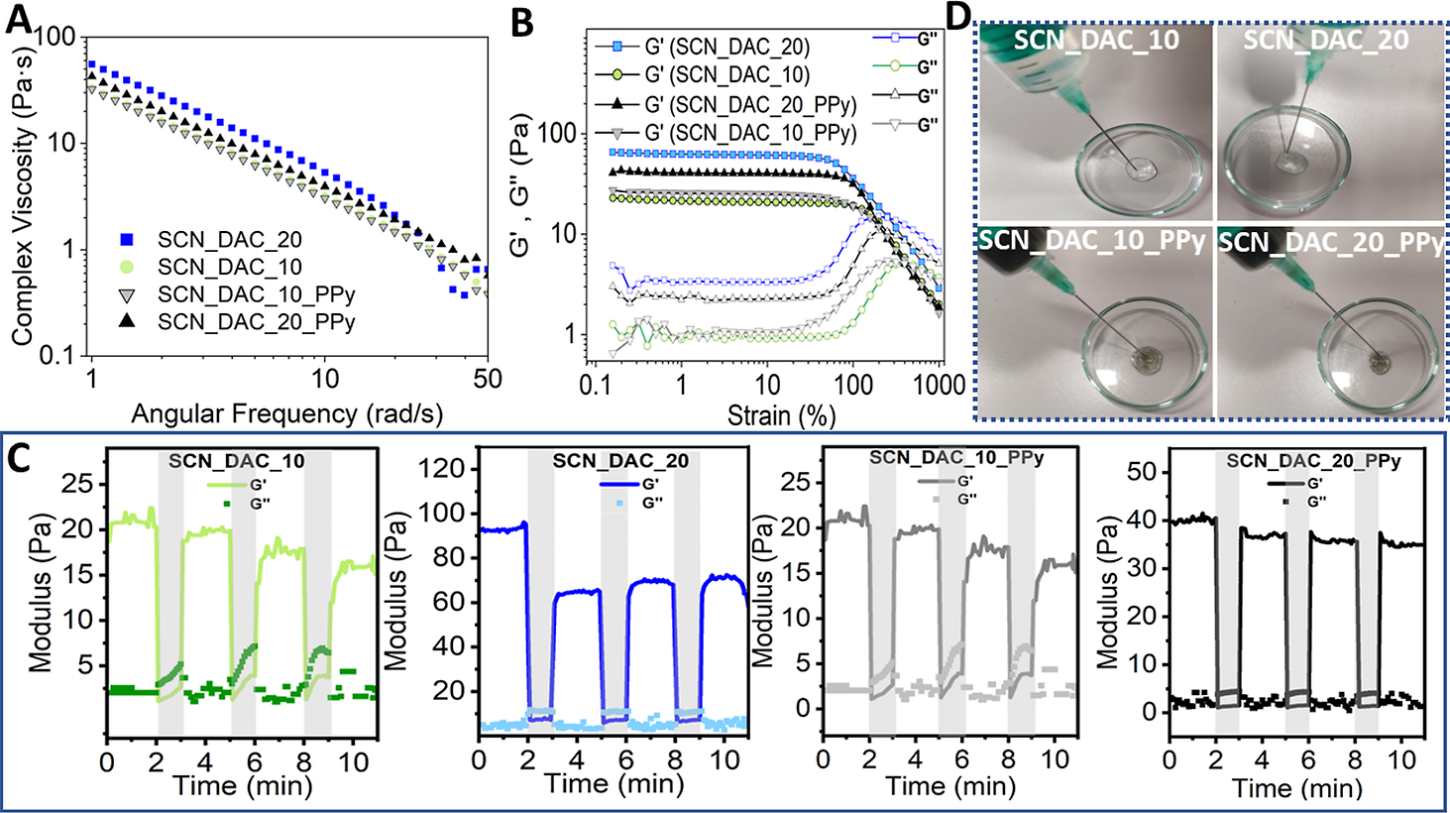
Part (A) presents the outcomes of viscosity dependence on angular frequency measurements. Part (B) shows the results of strain sweep tests for the prepared gels. Part (C) illustrates the time sweep rheology experiments with cyclic strain of the prepared gels, where shaded regions indicate high strain (1000%) and unshaded regions indicate low strain (0.2%). Part (D) shows the photographs of the prepared materials.

Furthermore, a strain sweep experiment was conducted with a range of strains from 0.1 to 1000% at 37 °C and a constant frequency of 1 Hz. As shown in Figure 4B, the prepared hydrogels exhibited linear viscoelastic behavior at low applied strains. In this linear viscoelastic region (LVE region), the storage modulus (*G*′) was higher than the loss modulus (*G*″), which is a typical behavior for hydrogels.^37^ However, at higher strain values (around 100%), the hydrogel structure was disrupted and all samples crossed the LVE region and transitioned to the nonlinear region.

Generally, four different types of behavior can be expected during LAOS (large-amplitude oscillatory shear) in the nonlinear region. Type I (strain-thinning) typically occurs when both *G*′ and *G*″ decrease due to reduced local resistance, caused by alignment of network segments with the flow field. Type II (strain-hardening) occurs when both *G*′ and *G*″ increase. Type III (weak strain overshoot) usually occurs when *G*′ decreases, while *G*″ initially increases and then decreases. The last Type IV (strong strain overshoot) occurs where both *G*′ and *G*″ initially increase and then decrease.^38^

The measured samples showed a rapid decrease in *G*′ in the nonlinear region, indicating stress on the hydrogel structure under increased strain amplitude, accompanied by a slightly increasing *G*″, which, after reaching a maximum, started to decrease (overshoot). The samples thus exhibit a type III nonlinear viscoelastic behavior. Upon reaching the crossover point, i.e., observing the maximum value of *G*″ (see Figure 4B), a balance between the formation and destruction of network connections occurs.^38^ Specifically, this behavior is known as weak strain hardening and is directly associated with the energy loss (dissipation) caused by the breakdown of the gel-like structure.^37^ After the overshoot of *G*″, the rate of decrease in *G*′ becomes more pronounced compared to the rate of decrease in *G*″ (see Figure 4B). This leads to a situation where, upon the crossover of *G*′ with *G*″, the deformation at *G*′ becomes smaller than at *G*″. This phenomenon results in the transition from a solid to a liquid due to the breakdown of the hydrogel network under sufficiently large deformations.

The SCN_DAC_20 hydrogel exhibited a crossover point at lower deformation amplitudes, specifically at 250%, compared to that of SCN_DAC_10, which had a crossover point at approximately 400%. This result can be attributed to the formation of a much stronger network in the former, which leads to smaller deformations. In PPy samples, the hydrogel network consists not only of Schiff bases between SCN and DAC but also of covalent bonds between DAC and PPy due to the aldol condensation reaction between the terminal pyrrole cycles of PPy and the aldehyde groups of DAC. The resulting network exhibits higher sensitivity to external forces, leading to faster initiation of deformation.^39^ Specifically, for sample SCN_DAC_20_PPy, the deformation amplitude was approximately 200%, significantly lower than in the case of SCN_DAC_20. Interestingly, a deformation amplitude of approximately 400% was measured for SCN_DAC_10_PPy, which is the same as that for SCN_DAC_10 (400%). The concentration of PPy particles was likely too low to significantly influence cross-linking in the bulk hydrogel. Observed effects can be attributed to the network topology defined by the reaction between PPy particles and DAC. While imine bonds between SCN and DAC are dynamic covalent bonds, the bonds between DAC and PPy are classical carbon–carbon bonds. The incorporation of PPy thus consumes a portion of the DAC cross-linker, which became permanently attached to the PPy particles through aldol condensation. This leads to a generally sparser bulk DAC/SCN network (lower *G*′), interspersed with DAC-modified PPy nanoparticles, serving as cross-linking “hotspots”. These regions of high cross-link concentrations in otherwise weaker networks may explain the reduced crossover strain (earlier structural breakdown) observed in SCN_DAC_20_PPy compared to SCN_DAC_20.

Next, cyclic strain tests were performed to evaluate the self-healing capability of the hydrogel (Figure 4C). The hydrogel network structures were disrupted when the strain was increased from 0.2% to 1000%, at which point the hydrogels behaved like liquids. Immediately after the restoration of the original strain value of 0.2%, the hydrogels underwent the sol–gel transition, which was completed in seconds. Despite a slight mechanical loss during the recovery process in some cases (SCN_DAC_20), all cyclic tests in the gel–sol transition were reversible during three alternately repeated cyclic tests. This demonstrated excellent self-healing behavior.

As an additional test, all hydrogels were successfully injected using a 21 G (0.8 mm) needle (see Figure 4D for photographs of the hydrogels during injection).

Based on the performed experiments, it can be concluded that all prepared hydrogel samples demonstrated significant viscosity reduction under stress and the ability to immediately and spontaneously recover, good injectability, and self-healing capability due to the reversible dynamic Schiff base interactions.

#### 3.2.2 Morphology and Electrical Conductivity

The morphology of the dried hydrogel samples was examined by using SEM analysis. After drying, the hydrogels formed sheet-like structures; representative SEM overview micrographs are provided in Figure S2 of the Supporting Information, with detailed images of individual sheets shown in Figure 5. While SCN_DAC_10 and SCN_DAC_20 hydrogels feature no significant micro- or nanoscale morphology, the SEM images of SCN_DAC_10_PPy and SCN_DAC_20_PPy samples show well-dispersed PPy particles within the hydrogels, which confirms our assumption regarding the good overall homogeneity of the investigated composite hydrogels.

**Figure 5:**
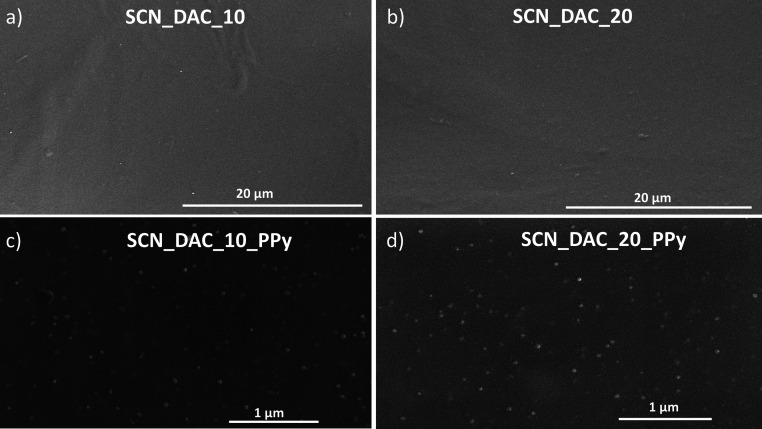
SEM images of dried (a) SCN_DAC_10, (b) SCN_DAC_20, (c) SCN_DAC_10_PPy, and (d) SCN_DAC_20_PPy samples.

Although the external electrical stimulation using prepared hydrogels was not the focus of this work, the electrical conductivity of the hydrogel samples was measured in their swollen state. All tested samples exhibited comparable conductivity values of approximately 0.3 ± 0.05 mS/cm. Notably, no statistically significant difference was observed between PPy-loaded and PPy-free samples, indicating that the measured conductivity is not dependent on the PPy colloid at the employed particle concentration. The concentration of PPy particles within the polymer matrix was likely too low to exceed the percolation threshold required for the formation of a continuous conductive network.

Nevertheless, the measured conductivity is biologically relevant as it falls well within the physiological conductivity range reported for skin components (10^–4^ to 2.6 mS/cm)^40^ and skeletal muscle.^41^ This match is crucial because skin and muscle are electrically sensitive tissues, and conductive materials have been shown to support physiological electrical signaling necessary for wound repair. Such materials promote cellular activities, including adhesion, proliferation, migration, and differentiation of electrically excitable cells such as fibroblasts, keratinocytes, nerve, bone, muscle, cardiac, and mesenchymal stem cells, even without an external electrical source.^40^ In our design, PPy primarily serves as a bioactive, redox-modulating component rather than as a means to create highly conductive scaffolds.

### 3.3 Biological Evaluation

The effect of all samples on cell viability, wound healing promotion, and anti-inflammatory action was evaluated in vitro.

#### 3.3.1 Cytocompatibility

The cytotoxicity of hydrogel extracts (0.1 g/mL) was evaluated according to ISO 10993-12 on the mouse embryonic fibroblast NIH/3T3 cell line; see Section S1 in the Supporting Information for more details. The parent extracts (100%) were diluted in culture medium to 75% and 50% concentration. As shown in Figure 6A, no hydrogel extract exhibited significant cytotoxicity, i.e. reduction of cell viability below 0.7, as defined by ISO 10993-5 (marked by a dashed line in Figure 6A).

**Figure 6:**
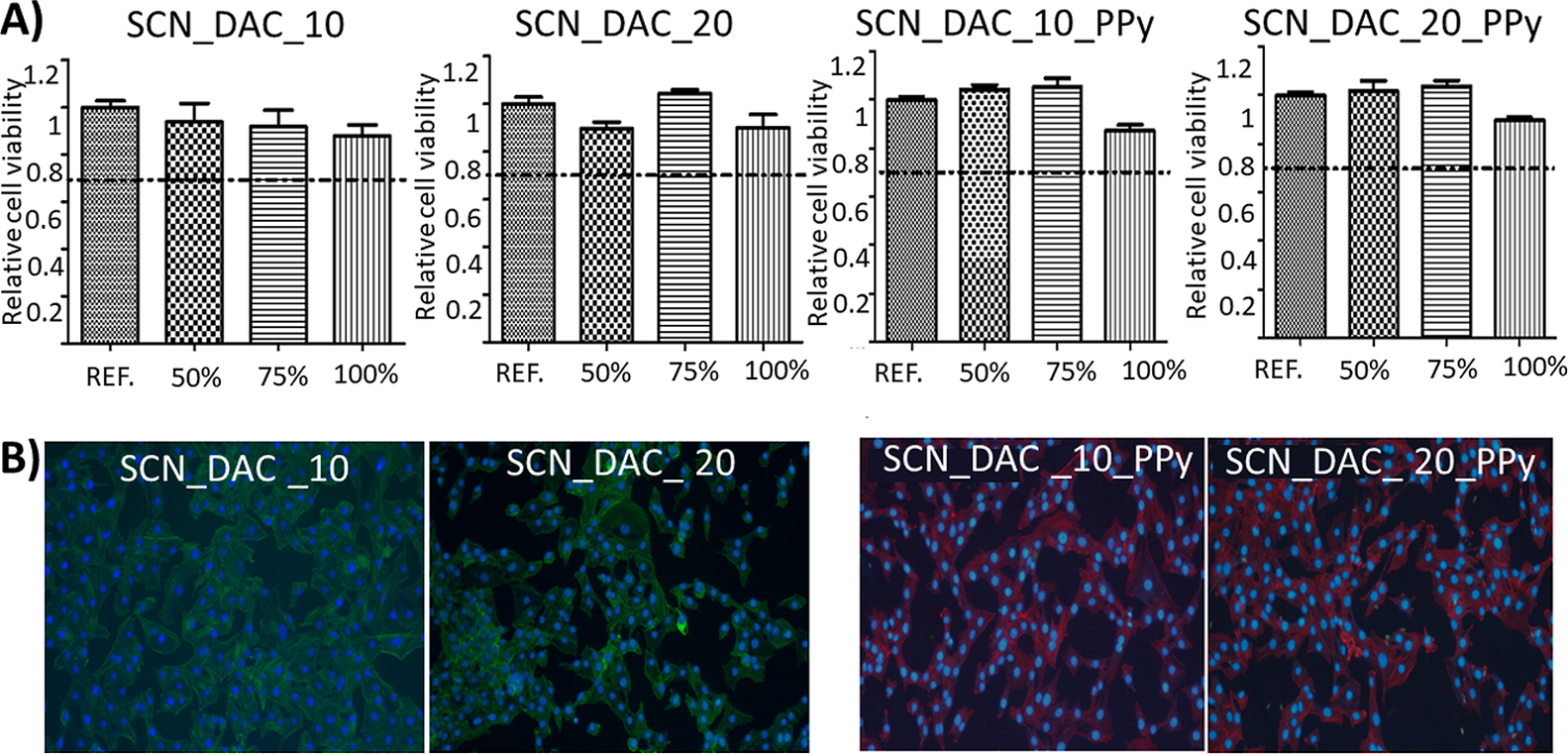
In Part (A), the cytotoxicity of hydrogel extracts at various concentrations (100%, 75%, and 50%) was assessed on the NIH/3T3 cell line according to ISO10993-12. The dashed line indicates the viability threshold as per EN ISO 10993-5 standards; viability below 0.7 indicates cytotoxic effects. Experiments were performed in quadruplicate. Percentages indicate the concentration of the hydrogel extract. Part (B) illustrates NIH/3T3 cell growth on culture plastic in the presence of the samples stained using Hoechst 33258 and actin green (SCN_DAC_10 and SCN_DAC_20) and actin red (PPy containing samples) to better distinguish between the hydrogel types. REF—culture plastic.

Additionally, NIH/3T3 cell growth in direct contact with hydrogels was studied for 48 h using confocal microscopy. Samples of neat hydrogels were stained with actin green, and samples containing PPy were stained with actin red to better distinguish the samples. No negative impact on cell growth was observed, with fibroblasts maintaining their physiological morphology (see Figure 6B). This indicates that the prepared hydrogels possess a good cytocompatibility.

#### 3.3.2 In Vitro Wound Healing Promotion

A scratch test was conducted to assess the impact of the prepared hydrogels on the migration of NIH/3T3 cells to simulate the wound healing process in vitro.^42^ The results show accelerated wound healing in the presence of all hydrogels compared to the reference (culture plastic); see Figure 7. The percentages of the wound areas that remained open after 10 h of incubation were 83 ± 7% for the reference, 65 ± 2% for the SCN_DAC_10 hydrogel, 63 ± 5% for the SCN_DAC_10_PPy, 65 ± 3% for the SCN_DAC_20 hydrogel, and 39 ± 2% for the SCN_DAC_20_PPy. Representative images of the wound of incubation with PPy-containing hydrogels and the reference can be found in the Supporting Information (Figure S3).

**Figure 7:**
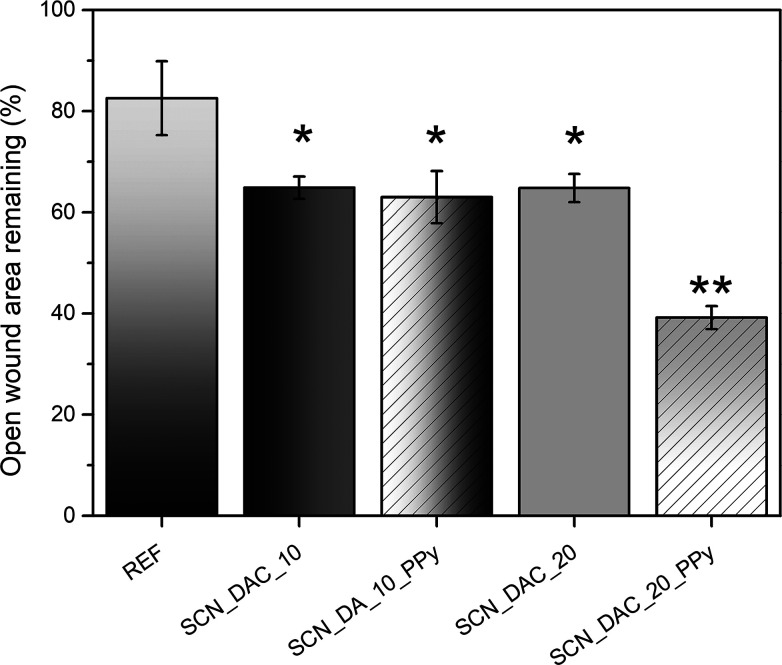
Percentage of the wound area remaining open after a 10 h incubation period (lower is better, REF—culture plastic). Experiments were performed in triplicate (*p < 0.05, **p < 0.01).

Interestingly, while the results of the composite SCN_DAC_10_PPy hydrogel were comparable to those of the SCN_DAC_10 hydrogel, the SCN_DAC_20_PPy sample accelerated wound healing significantly more than the neat SCN_DAC_20 hydrogel. The reason for this behavior is unclear. We can only speculate that it may be related to the difference in the viscoelastic properties. However, confirmation of this hypothesis would require dedicated biomechanistic studies, which are beyond the scope of this work.

#### 3.3.3 Influence of Hydrogels on Macrophage Response

Macrophages play a crucial role in all phases of wound healing, including inflammatory, proliferative, and remodeling phases. During the initial inflammatory phase, which lasts approximately 1–3 days after injury, pro-inflammatory macrophages, known as M1, predominate. These cells produce cytokines such as TNF-α, IL-6, and IL-1β, which promote inflammation while phagocytizing pathogens and removing cellular debris.^43^ Once the wound is cleaned, macrophages switch to an M2 phenotype, which is anti-inflammatory and promotes tissue repair. M2 macrophages are involved in angiogenesis, extracellular matrix synthesis, and the promotion of fibroblast and keratinocyte proliferation, all of which are key processes in tissue repair. Dysregulation of the balance between M1 and M2 macrophages can lead to chronic wounds.^43^

Note that for testing, the hydrogels were added to the culture media in varying amounts (from 0% to 60%). This was necessary to ensure the availability of necessary nutrients and oxygen for the cells in a 2D culture. This would not be a problem for living systems, where both nutrients and oxygen are provided by blood, but the obtained macrophage response can be somewhat reduced.

According to the MTT assay, the tested hydrogels had no cytotoxic effects on macrophages (Figure 8 left column), except for SCN_DAC_10_PPy, which showed a reduction in MTT activity to approximately 68% of the control at the highest concentration tested.

**Figure 8:**
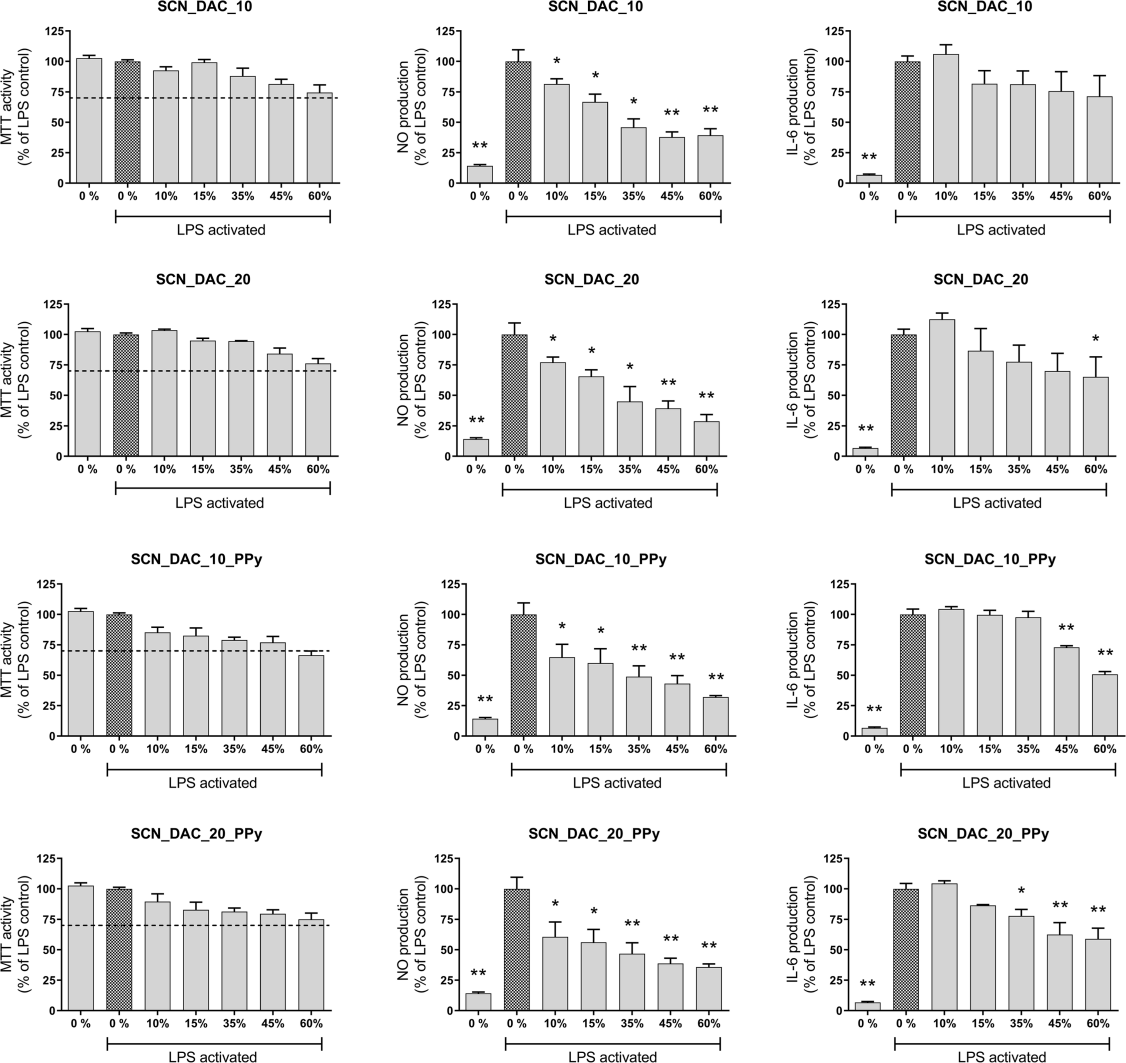
Effect of injectable hydrogels on mitochondrial activity (left column), NO (middle column), and IL-6 production (right column) of RAW264.7 macrophages. Results are obtained after 24 h for cells activated using 15 ng/mL of LPS (100% activation). Data were converted to a percentage of the relevant control and expressed as the mean ± SEM (*n* = 4). Statistical differences were tested by one-way ANOVA, which was followed by Dunnett’s multiple comparison test. *p*-Value for multiple comparisons was performed (*p < 0.05; **p < 0.01).

Next, the ability of hydrogels to influence pro-inflammatory markers, namely, NO and IL-6, typically associated with M1 macrophages, was tested. All four hydrogels reduced NO production compared to LPS-stimulated controls, indicating that both neat and PPy-containing composites can attenuate at least part of the pro-inflammatory response of macrophages. This is consistent with reported intrinsic anti-inflammatory and antioxidant properties of chitosan and its derivatives.^44^ However, only the PPy-containing hydrogels produced a statistically significant reduction in IL-6 secretion (Figure 8, middle and right columns), suggesting a PPy-dependent contribution to the modulation of cytokine expression. These data therefore support a model in which SCN and DAC provide a baseline anti-inflammatory microenvironment, while PPy further shifts macrophage responses toward a less pro-inflammatory phenotype.

The ability to modulate the inflammatory response in vitro suggests the potential of these hydrogels to promote the transition from a proinflammatory (M1) phase to a reparative (M2) phenotype of macrophages, which is crucial for efficient wound healing. Based on these results, it can be concluded that PPy-containing hydrogels exhibit anti-inflammatory properties and represent a promising material for the development of advanced wound dressings.

To further support the translational relevance of these hydrogels, the degradation behavior must be considered. Although long-term in vivo degradation studies are beyond the scope of this work, the biodegradation pathways can be predicted based on the established behavior of the individual components. The hydrogel matrix, composed of SCN and DAC, is expected to degrade primarily via enzymatic and hydrolytic degradation, which are well described in the literature.^45,46^ Regarding the PPy, our recent study on PVP-stabilized colloids has confirmed their low cytotoxicity and immunocompatibility.^26^ Furthermore, various in vivo investigations have demonstrated that neither bulk implants nor released micro- or nanoparticles induce acute or chronic (up to 6 months) systemic toxicity, organ damage, genotoxicity, or chronic inflammation.^47–49^ Therefore, degradation of the hydrogel is expected to release rather biologically stable and inert PPy particles, which should not induce any adverse effects.

## 4 Conclusions

This study focused on the preparation and characterization of injectable, self-healing composite hydrogels based on water-soluble half acetylated chitosan (SCN) and dialdehyde cellulose (DAC), incorporating polypyrrole (PPy) nanoparticles. The DAC served as a cross-linker for SCN (via a Schiff base reaction) and for PPy tethering (via an aldol condensation). The resulting hydrogels exhibited desirable shear-thinning properties, allowing for injection through syringe needles, and rapid self-healing capabilities due to dynamic covalent bonding. Importantly, all prepared hydrogels showed good biocompatibility with NIH/3T3 fibroblasts and RAW 264.7 macrophages and demonstrated significant anti-inflammatory activity by reducing NO and IL-6 production in LPS-stimulated macrophages. They also promoted in vitro wound healing by enhancing fibroblast migration, with the best overall results obtained for the SCN_DAC_20_PPy sample, i.e., that containing 20 mol % DAC relative to SCN and 5 wt % of PPy.

These findings suggest that injectable SCN_DAC_PPy hydrogels may be a promising starting point for the development of biomaterials for wound healing applications.

## Supplementary Material



## Notes

Sample characterization and instrumentation details; 1H NMR spectrum of source chitosan; overview SEM images of dried SCN/DAC/PPy hydrogels forming sheet-like structures; and representative images of the wound closure assay at 0 and 10 h (PDF)
