# Supplementary material for: Simultaneous Cross-Linking and Nanoparticle Anchoring by Dialdehyde Cellulose in Injectable Composite Chitosan/Polypyrrole Hydrogels
Source: ACS Appl Bio Mater. 2026 Feb 19;9(6):3038–49. doi: 10.1021/acsabm.5c02494 (PMC12997243; doi:10.1021/acsabm.5c02494)
Supplement: Supplementary file 1 [file mt5c02494_si_001.pdf]

Simultaneous Crosslinking and Nanoparticle Anchoring by Dialdehyde Cellulose in Injectable Composite Hydrogels

Supporting information

Monika Muchová [1], Lukáš Münster,[1] Roman Kolařík,[1] Zdenka Víchová,[1] Ondřej Vašíček,[2] Petr Humpolíček,[1,3] Jan Vícha,[1*]*

[1]Centre of Polymer Systems, Tomas Bata University in Zlín, tř. Tomáše Bati 5678, 760 01 Zlín, Czech Republic

[2]Institute of Biophysics of the Czech Academy of Sciences, Kralovopolská 135, 612 00 Brno, Czech Republic

[3] Department of Fat, Surfactant and Cosmetics Technology, Faculty of Technology, Tomas Bata University in Zlín, nám. T. G. Masaryka 5555, 760 01 Zlín, Czech Republic

Emails: m_muchova@utb.cz, jvicha@utb.cz

S1. Sample characterization and instrumentation

Evaluation of cytocompatibility

The cytotoxicity of hydrogel extracts was evaluated according to ISO 10993-12 using the mouse embryonic fibroblast cell line NIH/3T3 (ECACC 93061524, England). Extracts were prepared from 0.1 g of swollen hydrogel per 1 mL of culture medium. The culture medium contained DMEM with 10% bovine calf serum and 100 U/mL penicillin/streptomycin. Cells were pre-cultured at a concentration of 1 x 10^5 cells/ml of medium for 24 hours at 37°C and 5% CO_2 in a humidified atmosphere using the HERAcell 150i incubator from Thermo Scientific (USA). Subsequently, the culture medium was replaced with fresh medium, and the test samples were added to the pre-cultured cells. Cells cultured in pure medium served as the reference. Cell viability was quantified using MTT analysis, where absorbance was measured at 570 nm using the Infinite M200 Pro NanoQuant spectrophotometer from Tecan (Switzerland). Cytotoxicity was evaluated according to ISO 10993-5, with cell viability above 0.7 considered non-cytotoxic and values below 0.7 considered cytotoxic.

Further, cellular growth in direct contact with the hydrogels was evaluated. Cells were seeded onto culture plastic plates at a concentration of 1 × 10^5 cells/ml of medium and kept in an incubator at 37°C with 5% CO_2 in a humid environment. After 48 hours of growth, the cells were fixed and stained with Hoechst and ActinGreen™ 488. Cells cultured in a regular medium were used as the control. Cell morphology was examined using the Olympus Fluoview FV3000 confocal microscope (Olympus, Japan).

In vitro wound healing promotion

The scratch assay was performed to evaluate the wound-healing capacity of the NIH/3T3 mouse embryonic fibroblast cell line[1]. Cells were seeded onto tissue culture dishes at a concentration of 2 x 10^5 cells/ml of media and incubated at 37°C with 5% CO_2 in a humid atmosphere until they formed a confluent monolayer. Then, a scratch was created on the monolayer using a sterile pipette tip, and any remaining cells were washed away with PBS. Images of the scratch were captured, and the dishes were replenished with fresh medium containing the test samples. They were further incubated at 37°C with 5% CO_2 in a humid environment. Cell images were taken at 0h and 10h using a phase-contrast microscope Olympus IX81 (Olympus, Japan). T-Scratch software (CSElab, Switzerland) was used for analysis, and the results are presented as the percentage of remaining open wound areas at 0 and 10 hours. Results are expressed as mean ± SEM (n = 3).

Immunomodulatory activity

Viability of RAW264.7 cells - The viability of murine peritoneal macrophages (RAW 264.7 cell line, European Collection of Authenticated Cell Culture, UK) was assessed in the presence of hydrogels alone or in combination with lipopolysaccharide (LPS), a known macrophage activator. Viability was determined by measuring the number of metabolically active cells using the MTT assay or similar tetrazolium salts in whole-cell lysates, as described by Moosova et al. (2019).[2] RAW264.7 cells were cultured in DMEM medium supplemented with 10% heat-inactivated, low-endotoxin fetal bovine serum (FBS) and 1% penicillin-streptomycin solution. RAW 264.7 cells were cultured in 24-well flat-bottom plates at 2.5×10^5 cells/well, together with SCN_DAC_10, SCN_DAC_20, SCN_DAC_10_PPY, or SCN_DAC_20_PPy injectable hydrogels. LPS (15 ng/mL, *E. coli* O26:B6, Sigma-Aldrich Co.) was added to the macrophages along with the tested hydrogels. Injectable hydrogels were mixed with the medium in 60%, 45%, 35%, 15%, and 10% concentrations to provide necessary nutrients for the cells. These hydrogel concentrations correspond to PPy concentrations of 1%, 0.75%, 0.5%, 0.25%, and 0.17% in the mixture. The total volume in each well was 500 µL. Cells were incubated for 24 h at 37°C in a humidified atmosphere containing 5% CO_2 and 95% air. At the end of the incubation period, cell

viability was measured using the MTT assay. Absorbance was recorded at 570 nm using a SPECTRA Sunrise microplate reader (Tecan, Mannedorf, Switzerland). Cytotoxicity was evaluated according to ISO 10993-5 protocol (2019), where the viability of cells in the reference sample was set as 1 (100% viability). Viability higher than 70% was interpreted as the absence of cytotoxicity, while values lower than 70% indicated cytotoxic effects.

Nitric oxide (NO) and interleukin 6 (IL-6) production by murine macrophages.

Changes in NO production were measured indirectly by the accumulation of nitrites (the end product of NO metabolism) in the medium by using Griess reagent (Sigma-Aldrich Co.), according to the method described previously (Vasicek et al., 2020).[3] To determine the effect of the tested injectable hydrogels, macrophages were incubated in combination with the LPS. The incubation conditions were the same as for the viability detection using the MTT method described above. At the end of incubation, the supernatants were collected by centrifugation at 16,000 g at 4 °C for 5 minutes, and 80 µL of each sample was mixed with an equal volume of the Griess reagent in a 96-well plate. The reaction mixtures were incubated at room temperature for 30 min in the dark, and the absorbance was read at 540 nm. A standard curve of 0–52 µM $NaNO_2$ was prepared and used for calculations.

The concentration of pro-inflammatory cytokine IL-6 produced by RAW264.7 cells in the cultivation medium was determined after 24 h of exposure to tested injectable hydrogels and LPS by commercially available immunoassays (Mouse IL-6 DuoSet, R&D Systems). The assays were performed according to the manufacturer's instructions.

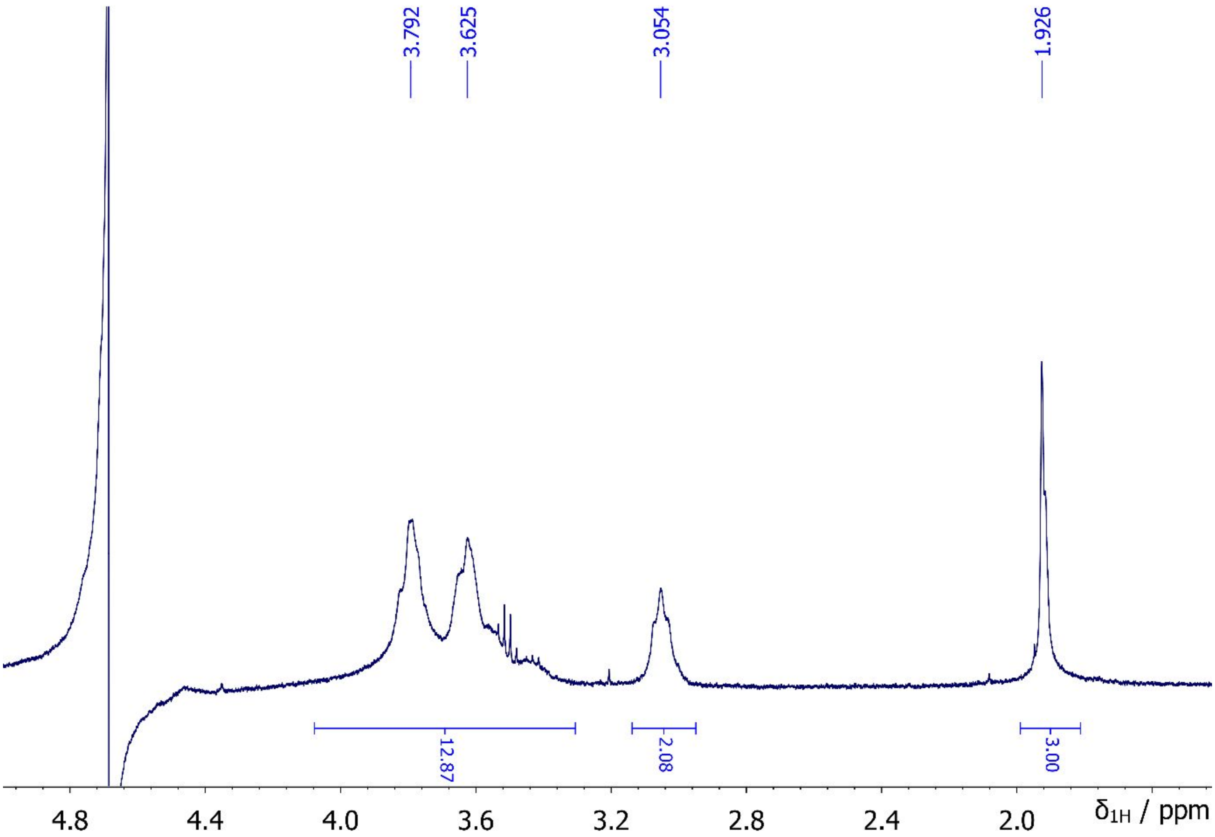

Fig. S1: ^{1}H NMR spectrum of source chitosan, 0.1 M DCl in D_2O, 298K.

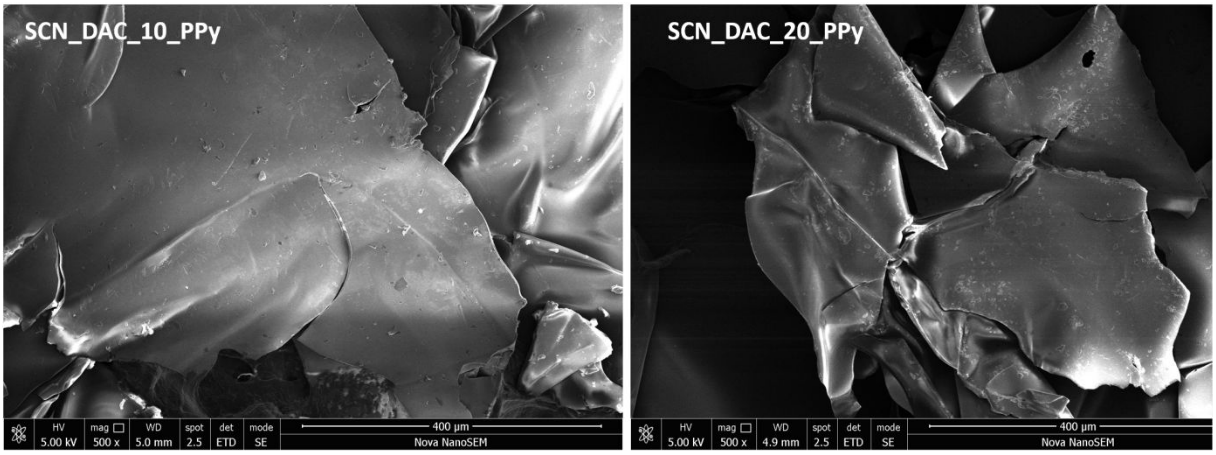

Fig. S2: Overview SEM images of dried SCN_DAC_10_PPy, and SCN_DAC_20_PPy samples forming sheet-like structures.

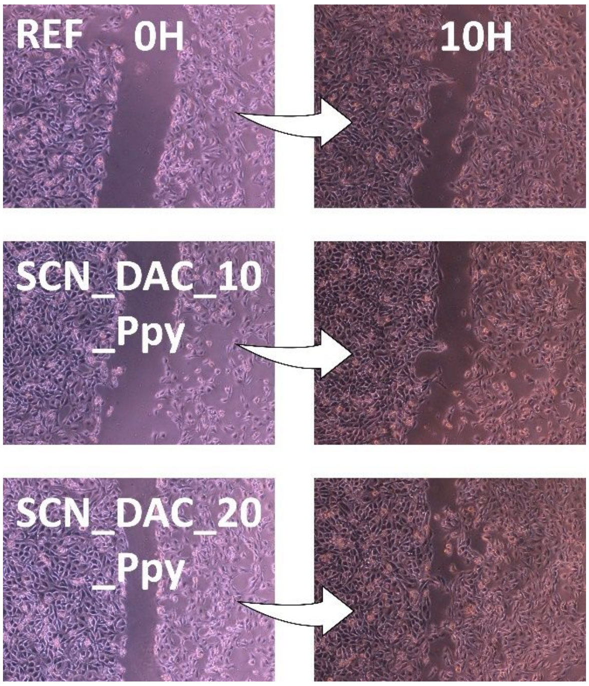

Fig. S3: Representative images of the wound at the initial 0 hours (left) and after 10 hours (right) of incubation with PPy-containing hydrogels and the reference

[1] C.-C. Liang, A.Y. Park, J.-L. Guan, In vitro scratch assay: a convenient and inexpensive method for analysis of cell migration in vitro, Nat. Protoc. 2 (2007) 329–333. https://doi.org/10.1038/nprot.2007.30.
[2] Z. Moosova, M. Pekarova, L.S. Sindlerova, O. Vasicek, L. Kubala, L. Blaha, O. Adamovsky, Immunomodulatory effects of cyanobacterial toxin cylindrospermopsin on innate immune cells, Chemosphere 226 (2019) 439–446. https://doi.org/10.1016/j.chemosphere.2019.03.143.
[3] O. Vasicek, D. Rubanova, B. Chytkova, L. Kubala, Natural pseurotins inhibit proliferation and inflammatory responses through the inactivation of STAT signaling pathways in macrophages, Food Chem. Toxicol. 141 (2020) 111348. https://doi.org/10.1016/j.fct.2020.111348.
